# Automated Software Acceleration in Programmable Logic for an Efficient NFFT Algorithm Implementation: A Case Study

**DOI:** 10.3390/s17040694

**Published:** 2017-03-28

**Authors:** Manuel Rodríguez, Eduardo Magdaleno, Fernando Pérez, Cristhian García

**Affiliations:** 1 Department of Industrial Engineering, Universidad de La Laguna, 38203 San Cristóbal de La Laguna, Spain; emagcas@ull.es (E.M.); cristhianj.gc@gmail.com (C.G.); 2 Department of Computer Engineering and Systems, Universidad de La Laguna, 38203 San Cristóbal de La Laguna, Spain; fdoperez@ull.edu.es

**Keywords:** NFFT, SDSoC, software acceleration, Zynq, parallelism techniques

## Abstract

Non-equispaced Fast Fourier transform (NFFT) is a very important algorithm in several technological and scientific areas such as synthetic aperture radar, computational photography, medical imaging, telecommunications, seismic analysis and so on. However, its computation complexity is high. In this paper, we describe an efficient NFFT implementation with a hardware coprocessor using an All-Programmable System-on-Chip (APSoC). This is a hybrid device that employs an Advanced RISC Machine (ARM) as Processing System with Programmable Logic for high-performance digital signal processing through parallelism and pipeline techniques. The algorithm has been coded in C language with pragma directives to optimize the architecture of the system. We have used the very novel Software Develop System-on-Chip (SDSoC) evelopment tool that simplifies the interface and partitioning between hardware and software. This provides shorter development cycles and iterative improvements by exploring several architectures of the global system. The computational results shows that hardware acceleration significantly outperformed the software based implementation.

## 1. Introduction

Fourier methods provide an important tool in wide areas of applied mathematics and physics. They were originally designed by Fourier in 1807 to model functions with trigonometric series, and, nowadays, they have become one of the fundamental techniques in digital signal and image processing. The most important step to make Fourier methods practical was the development by Cooley and Tukey [1] of the Fast Fourier Transform (FFT) for the fast computation of the discrete Fourier transform (DFT). The FFT needs only O(NlogN) operations instead of the O($N^{2}$) arithmetical operations for its direct computation.

The Fast Fourier Transform (FFT) has been used in almost every area of modern science including astronomy, digital signal processing, image and video processing or medicine. However, the FFT requires the input signal to be sampled on an equally spaced grid, which introduces an important limitation. There are several areas such as synthetic aperture radar (SAR) [2], computational photography [3], medical imaging [4], telecommunications [5], seismic analysis [6] or smart sensors like plenoptic cameras [7,8], where data acquisition is carried out from an irregular and non-equispaced sampling. Problems arising from these areas caused the need of a fast Fourier transform for non-equispaced nodes (NFFT).

The first NFFT methods were introduced in the field of digital signal and image processing in [9,10]. A popular technique for the NFFT is the gridding [11,12] method, which provides low computation complexity with controllable approximation error. The basic idea of gridding is to re-sample the non-equispaced input sampled data on a regular grid by performing an interpolation using linear combinations of translates of a window function φ having good localization in the time and frequency domain. Then, the inverse FFT is used to reconstruct the target signal.

The first results deriving the connection between the computational complexity of the algorithm and desired accuracy were provided by Dutt and Rokhlin [13] for the Gaussian window function φ and by Beylkin [14] for B-splines. Subsequent work [8,12,15] unified both approaches and provided consistent error estimates based on the split of the overall error in an aliasing error and a truncation error. These estimations suggested the use of window functions with better properties. In particular, Kaiser–Bessel functions [16] and powers of the sinc-function [17] provide good results. There are open source implementations for the NFFT. One is the C subroutine library of Kunis and Potts [18]; another is the Matlab 7 toolbox by Fessler and Sutton [19].

Computing paradigms for high performance applications have been based in the use of one or several CPUs in standard computer architecture. However, as the demand for computing power increased, other solutions like Field Programmable Gates Array and Graphics Processor Unit (FPGAs and GPUs) save emerged as the platforms of choice for computationally demanding applications. Traditionally, FPGAs have been used mostly for fixed-point applications, while GPUs have mostly been used for applications requiring floating-point computations. Recently, the computational and memory resources on FPGAs have massively increased and now it is common to adopt FPGAs for floating-point applications as well [20,21]. In this paper, we describe a FPGA implementation for the NFFT transform. 

This implementation is necessary for example to obtain the focal stack from a 4D lightfield plenoptic sensor using Fourier Slice techniques. At the present time, there is a growing interest in the development of plenoptic cameras capable of processing in real time the 4D lightfield. For this purpose, we are developing an embedded low power consumption real-time system for plenoptic cameras based on FPGA. Nevertheless, system development in FPGAs currently leads to complex analyses, high development time, costly debugging and difficult implementation [6,7]. These reasons have motivated us to explore new tools that help to obtain shorter development time. 

We have used the very novel SDSoC development tool that simplifies interface and partitioning between hardware and software. There are other approaches that implement the NFFT algorithm with FPGAs. In these cases, the algorithm is written in hardware description language (HDL). This causes the development stages to be slow because they have to take into account low-abstraction level considerations such as memory management. It also needs to use proprietary cores such as the Xilinx floating point of the Xilinx core Generator [22] (Xilinx, Inc., San Jose, CA, USA). 

This paper presents the experiences encountered using the SDSoC High Level Synthesis (HLS) tools, in order to design and implement an accelerated and efficient NFFT algorithm. The algorithm was targeted for implementation on ZedBoard (Digilent Inc., Pullman, WA, USA). It was coded using C language with pragmas to optimize the architecture of the system. Thus, we do not need to concentrate in the details of the low abstraction level and we can easily explore several architectural solutions in a short period of time.

This paper is structured in six sections including this Introduction. First, we describe the theoretical background of the NFFT algorithm and the problem specification. Then, Section 3 describes the technology used in order to accelerate the algorithm using programmable logic, and Section 4 describes how to implement the algorithm using Zynq. Section 5 explains the experimental results, and, finally, conclusions and future work are presented in Section 6.

## 2. The Non-Equispaced Fast Fourier Transform (NFFT)

In this section, we will introduce the Non-equispaced Fast Fourier Transform (NFFT), discuss its properties and detail its computation in the algorithmic level. The NFFT efficiently computes approximations of sums:

(1) $$f\left(x_{j}\right)=\overset{\frac{N}{2}-1}{\underset{k=-N/2}{\sum }}{\hat{f}}_{k}e^{-2\pi ikx_{j}},j=1,\ldots ,M$$

at arbitrary nodes $x_{j}\in \left[-1/2,1/2\right).$

Let *N* be an even number and *I_N_* = {$-\frac{N}{2},\ldots \frac{N}{2}-1\}.$ The computation of the NFFT is based on the approximation of the trigonometric polynomial

(2) $$\left(x\right)=\sum_{k\in I_{N}}{\hat{f}}_{k}e^{-2\pi ikx}.$$

Let φ be an even window function so that its periodic version $\tilde{\varphi }=\sum_{r\in \mathbb{Z}}\varphi \left(x+r\right)$ has an absolute convergent Fourier series:

(3) $$\tilde{\varphi }\left(x\right)=\sum_{k\in \mathbb{Z}}c_{k}\left(\tilde{\varphi }\right)e^{-2\pi ikx},$$

with Fourier coefficients:

(4) $$c_{k}\left(\tilde{\varphi }\right)=\int_{-\frac{1}{2}}^{\frac{1}{2}}\tilde{\varphi }\left(x\right)e^{2\pi ikx}dx=\int_{-\infty }^{\infty }\varphi \left(x\right)e^{2\pi ikx}dx,k\in \mathbb{Z}.$$

Let σ>1 be an oversampling factor and let n=σN. The NFFT tries to approximate f with:

(5) $$g\left(x\right)=\sum_{l\in I_{n}}g_{l}\tilde{\varphi }\left(x-\frac{l}{n}\right).$$

To define the $g_{l}$, we change into the frequency domain obtaining:

(6) $$g\left(x\right)=\underset{k\in \mathbb{Z}}{\sum }{\hat{g}}_{k}c_{k}\left(\tilde{\varphi }\right)e^{-2\pi ikx}=\;\sum_{k\in I_{n}}{\hat{g}}_{k}c_{k}\left(\tilde{\varphi }\right)e^{-2\pi ikx}+\sum_{r\in \mathbb{Z}-\left\{0\right\}}\sum_{k=-n}^{n}{\hat{g}}_{k}c_{k+nr}\left(\tilde{\varphi }\right)e^{-2\pi i\left(k+nr\right)x},$$

with discrete Fourier coefficients:

(7) $${\hat{g}}_{k}=\sum_{l\in I_{n}}g_{l}e^{2\pi ikl/n},\;g_{l}=\frac{1}{n}\sum_{l\in I_{n}}{\hat{g}}_{k}e^{-2\pi ikl/n}.$$

Supposing that the Fourier coefficients become sufficiently small for $\left|k\right|\geq n-\frac{N}{2}$ and $c_{k}\left(\tilde{\varphi }\right)\neq 0$ for $k\in I_{n}$; then, comparing Equations (6) and (2) suggests the relation:

(8) $${\hat{g}}_{k}={\hat{g}}_{k+nr}=\{\begin{array}{cc} {\hat{f}}_{k}/c_{k}\left(\tilde{\varphi }\right) & k\in I_{N}\\ 0 & k\in I_{n}-I_{N} \end{array}$$

for r∈ℤ. Then, the values of $g_{l}$ can be obtained from Equation (7) by an FFT of size *n*. This approximation causes an aliasing error.

Now, if we assume also that φ is well-localized in the time domain, then it can be approximated by a function:

(9) $$\psi \left(x\right)=\varphi \left(x\right)\chi_{\left[-\frac{m}{n},\frac{m}{n}\right]}\left(x\right),$$

with support $\psi =\left[-\frac{m}{n},\frac{m}{n}\right]$ and cut-off parameter m∈ℕ and m≪n.

Using its periodic version $\tilde{\psi }$ and the index set: $I_{n,m}\left(x_{j}\right)=\left\{l\in I_{n},\;nx_{j}-m\leq l\leq nx_{j}+m\right\},$ an approximation to g(x) is defined as:

(10) $$f\left(x_{j}\right)\approx g\left(x_{j}\right)\approx \sum_{I_{n,m}\left(x_{j}\right)}g_{l}\tilde{\psi }\left(x_{j}-\frac{l}{n}\right).$$

For a fixed $x_{j},$ the preceding sum contains at most 2m+1 summands. This approximation causes a truncation error. In summary, the NFFT approximates $f\left(x_{j}\right)$ by means of the following steps:

**NFFT algorithm**
Inputs:
N complex numbers ${\hat{f}}_{k},\;k\in I_{N}$, M arbitrary nodes $x_{j}\in \left[-1/2,1/2\right).$
Parameters:
Window function φ, Oversampling factor σ, Cut-off parameter m.
Output:
M function evaluations $f\left(x_{j}\right).$

**Step 1 (Deconvolution)**
  For $k\in I_{N}$ compute:
  (11) $${\hat{g}}_{k}=\frac{{\hat{f}}_{k}}{c_{k}\left(\tilde{\varphi }\right)}.$$
**Step 2 (Fast Fourier Transform)**
  For $l\in I_{n}$ compute the FFT:
  (12) $$g_{l}=\frac{1}{n}\underset{l\in I_{n}}{\sum }{\hat{g}}_{k}e^{-2\pi ikl/n},\;n=\sigma N.$$
**Step 3 (Convolution)**
  For j=0,…,M−1 compute:
  (13) $$f_{j}=\underset{I_{n,m}\left(x_{j}\right)}{\sum }g_{l}\tilde{\psi }\left(x_{j}-\frac{l}{n}\right).$$

The analysis of the algorithm shows that the computational complexity is O(nlogn+mM). To keep the approximation error small, several window functions φ with good localization in time and frequency domain were proposed like the *Gaussian* [8,12], B-*splines* [13,14], *sinc functions* [18] or *Kaiser–Bessel functions* [23]. A detailed analysis of the approximation errors can be found in the corresponding papers. In general, for a fixed σ, the approximation error decays exponentially in the cut-off error *m*.

## 3. Description of Technology

In this section, we will describe the Zynq hardware platform (Xilinx Inc.) used on the implementation of the algorithm. In addition, we will describe the novel development tool (SDSoC) that we have used in the design stages. 

### 3.1. The Hardware Platform

The prototype has been implemented into the Zedboard development board [24]. The most relevant of this development board is the Zynq-7020 device [25]. Basically, Zynq combines a dual-core ARM cortex-A9 processor [26] with traditional FPGA logic fabric. Xilinx calls it an APSoC (All Programmable System-on-Chip) [27]. 

**Figure 1:**
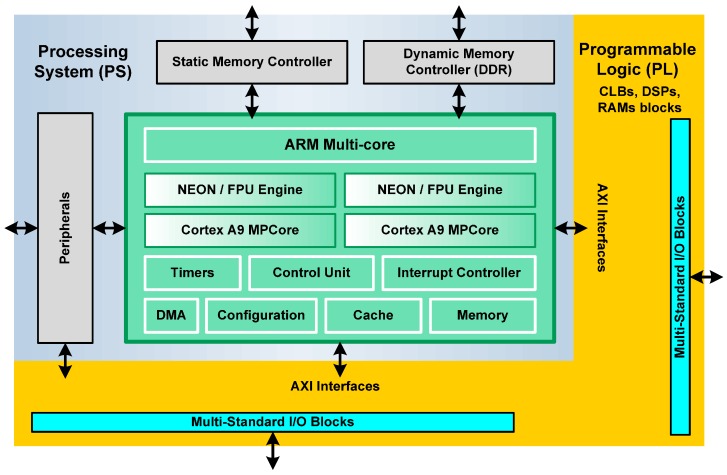
Simplified architecture model of a 7020 Zynq device.

The general architecture of a Zynq device comprises two sections: the Processing System (PS) and the Programmable Logic (PL) (Figure 1). Both can be used independently or together. 

The Processing System is a hardware dual-core ARM Cortex processor, an alternative to soft processors like PicoBlaze, MicroBlaze from Xilinx Inc. etc. [28,29]. Soft processors are implemented in the logic fabric of the FPGA, resulting in a very flexible component. On the other hand, hard processors can achieve considerably higher performance. Each ARM core has a Neon Processing Engine and a Floating Point Unit, in order to realize computational tasks. The Control Unit assumes several tasks related to the interface between processors and cache memories. In addition, this component manages transactions that take place between the Processing System and Programmable Logic. The ARM multi-core operates to 866 MHz [27]. 

**Figure 2:**
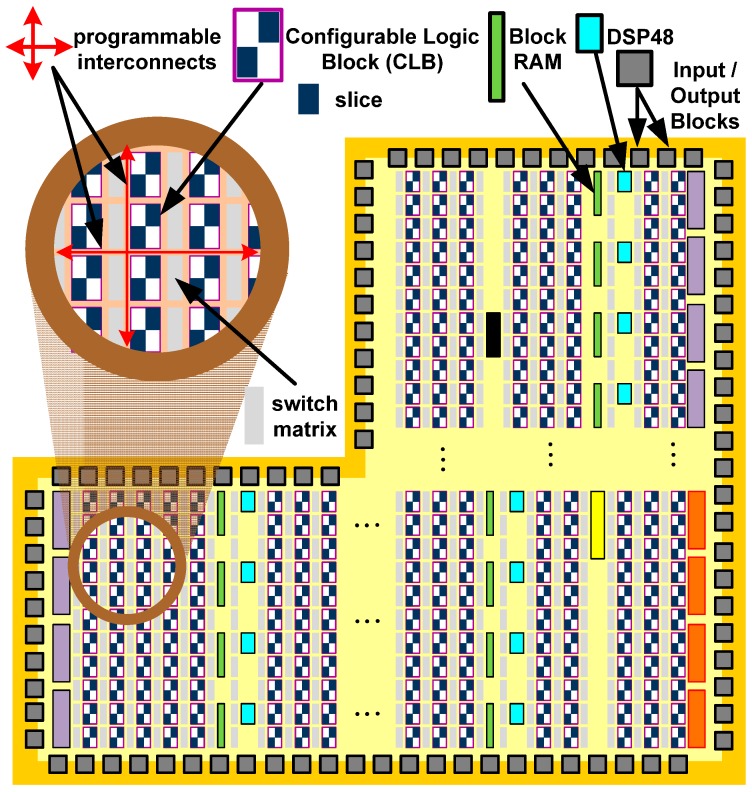
The programmable logic of the Zynq-7020 device and its constituent elements.

The Programmable Logic of the Zynq-7020 device is based on the Artix-7 Xilinx FPGA fabric and it is depicted in Figure 2. This logic fabric consists of an array of Configurable Logic Blocks (CLBs) which are composed of two slices. These elements are connected to other similar blocks via programmable interconnects and switch matrices. Inside of a slice, there are four Lookup Tables (6-LUT) capable of implementing a logic function up to six inputs, a small memory (named distributed-memory) or a shift register. Each slice also has eight flip-flops to implement sequential circuits. The Zynq-7020 has 53,200-LUT and 106,400 flip-flops [25].

In addition to the general fabric, the Programmable Logic has several special purpose components. The most frequently used are Block RAMs for dense memory requirements and DSP48 slices for high speed arithmetic. The last component is comprised of a pre-adder/subtractor, multiplier, and post-adder/subtractor. All of these resources are integrated into the logic array in a column arrangement and embedded into the logic fabric. The Zynq-7020 on Zedboard has 140 × 36 Kb Block RAMs and 220 DSP48 slices (18 × 25 bit) [27].

### 3.2. Software Tools and Design Methodology

As mentioned before, the Zynq device comprises a Processing System and Programmable Logic. Therefore, it can potentially replace two devices with the advantages of these two technologies. The PS component can support software routines, GUIs or an operating system (OS) with control tasks and applications (including data processing), and the PL component can be used for implementing algorithms that are inherently parallel in nature. Examples of these implementations are algorithms for data processing like the NFFT, where mathematical operations are performed on a large number of samples simultaneously, and where software implementations result in a bottleneck. 

**Figure 3:**
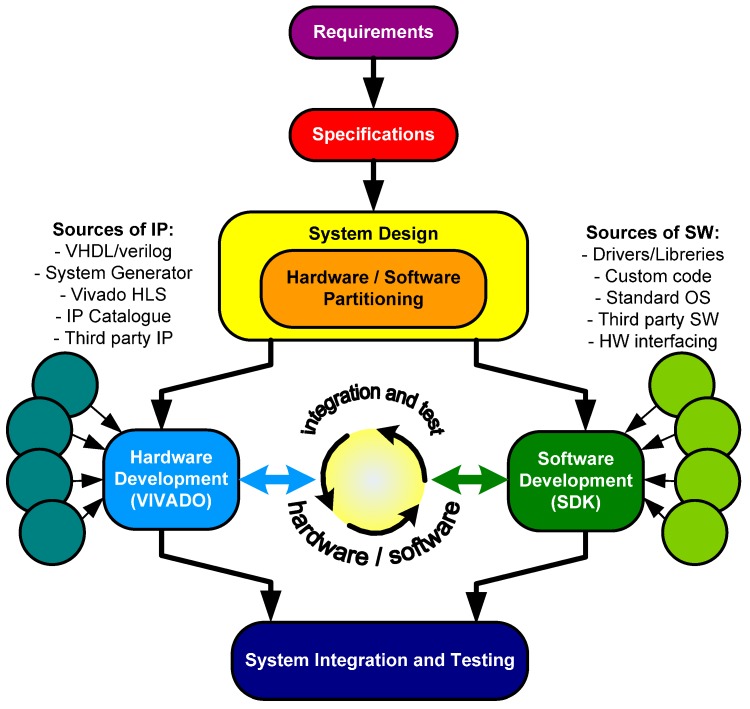
The traditional hardware/software co-design flow.

At the beginning, the design flow for Zynq devices was like the traditional Hardware/Sotfware HW/SW co-design using soft-cores such as the MicroBlaze (Figure 3) [30]. First, the engineer had to identify the individual subsystems or functional tasks. Then, the design had to be appropriately partitioned into hardware and software. Finally, the necessary communication between different parts of the system had to be defined. The Vivado and Software Development Tool SDK tools (Xilinx Inc.) are the elements for hardware and software system design. Vivado is used for creating the hardware system component and also includes facilities for integrating IPs (including Very High Description Language VHDL/Verilog modules, System Generator, third party IPs and C-level descriptions through Vivado High-Level Synthesis). SDK is a software design suite that includes driver support for IPs, Gnu C language Compiler (GCC) library support for ARM and Neon using C and C++ languages and tools for debugging and profiling [31,32]. 

Vivado provides the coupling between the Zynq processor and the implemented modules in programmable logic via AXI interfaces [29]. Although it is very important to define the partition between hardware and software at the beginning of the design phase, the engineer usually has to refine this part of the project due to the complexity of the task, and it must be gradually improved. Unfortunately, these design changes are undesirable because they slow down significantly the project development. Recently, Xilinx has released a new tool that automatizes even further the design phase using the SDSoC [33] hybrid technology. This tool simplifies interface and partitioning between hardware and software. It causes shorter development cycles and iterative improvements can be made at early stages of development without the need to build the hardware. Using only software code in C or C++, it generates hardware code to implement in the programmable logic of Zynq device. This allows the engineer to test several alternative solutions fast and easily. 

**Figure 4:**
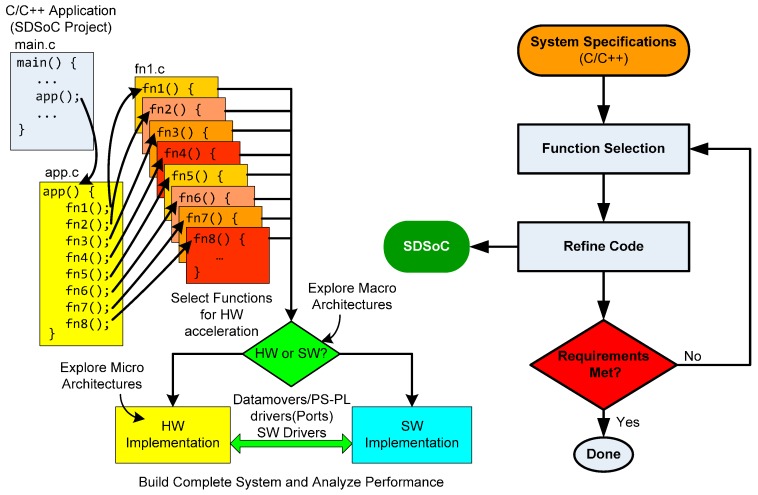
Flow design using SDSoC.

The design flow using SDSoC is depicted in Figure 4. In an SDSoC project, the user has to select which functions must be implemented in hardware. The only requirement is that these functions have to be coded in its own C-file. The platform can estimate which function or functions are suitable or are the best candidates for a hardware acceleration implementation. This constitutes an exploration of macro architectures. Then, each function suitable to HW is refined to obtain the optimized description (analysis of micro architectures). In this step, pragmas or directives are introduced (this element is explained in the next section). SDSoC software development includes Vivado for Zynq devices, in order to analyze the generated hardware schema. In fact, Vivado HLS is used as a Zynq PL cross-compiler in SDSoC. The platform also includes a profiling system to easily calculate the performance in acceleration and resources of each architectural solution.

**Figure 5:**
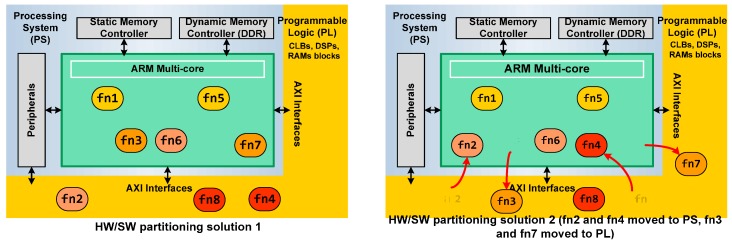
Example of two function selections to implement the same application.

Figure 5 depicts a general example of two architecture solutions for a design. The system application has eight functions suitable to HW acceleration. On the left side of the figure, HW/SW partitioning, where functions 2, 4 and 8 are built in the Programmable Logic of Zynq, can be shown, and the rest are executed as SW in the ARM. On the right side, other HW/SW partitioning is explored. In this case, functions 2 and 4 are passed to the ARM and functions 3 and 7 are implemented in HW. The communication between the Processing System and the Programmable Logic is automatized by SDSoC using the AXI interfaces. 

## 4. C Code Function to Hardware Module

**Figure 6:**
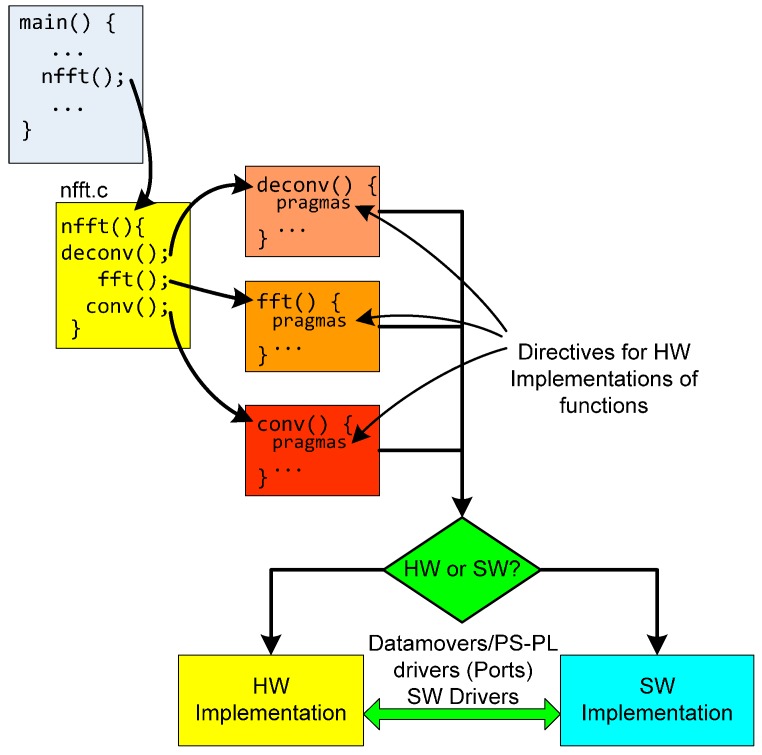
Flow design for NFFT algorithm implementation in Zynq.

The SDSoC has a C/C++ full system optimizing compiler, a system level profiler, automated software acceleration in programmable logic and automated system connectivity generation It supports Bare metal, Linux and Free Real Time Operating System (FreeRTO) as target OS. To implement the code, we evaluated the possibility of using the open source C code of the NFFT libraries. Nevertheless, we discarded this possibility due to the complex coding of the algorithm in the libraries and the original data types that were not supported by the SDSoC tool. Therefore, we implemented the algorithm described in Section 2. NFFT algorithm is formed by three modules of functions: data deconvolution, FFT and convolution modules and its input and output interfaces (Figure 6).

Each module was implemented in C language as three independent functions. The FFT transform that we have used is based on the numeric recipes in C code with an SDSoC type. The other two functions were coded specifically for this implementation. The data input/output are float complex data. We have implemented our own complex data type to be compatible with a correct hardware implementation instead of the complex type from C language.

We tried with different designs by just toggling the target of that function, i.e., hardware or software. In first place, the NFFT code was executed on the PS (ARM processor from Zinc-FPGA system) to evaluate the functionality and performance of each module and the whole algorithm. 

To explore which part of code is more time-consuming, we selected in the SDSoC Tool that each module would be implemented in hardware while the other modules run on the ARM. We repeated this action three times for each module. We also tested all modules of the NFFT algorithm in hardware implementation using SDSoC. The SDSoC linker creates an SD card image of the application solution in Linux environment. The system that resulted from this approach was downloaded into the Zync board. A Telnet connection allowed us to run the code with different input configurations parameters.

**Figure 7:**
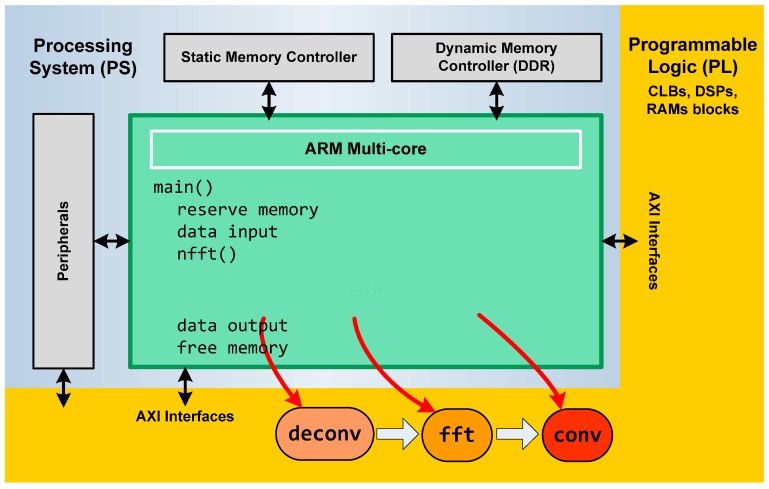
NFFT HW/SW C -code acceleration using Zynq. SDSoC is a flexible tool that permits us to select independently one, two or three functions of our algorithm to be implemented in HW.

**Figure 8:**
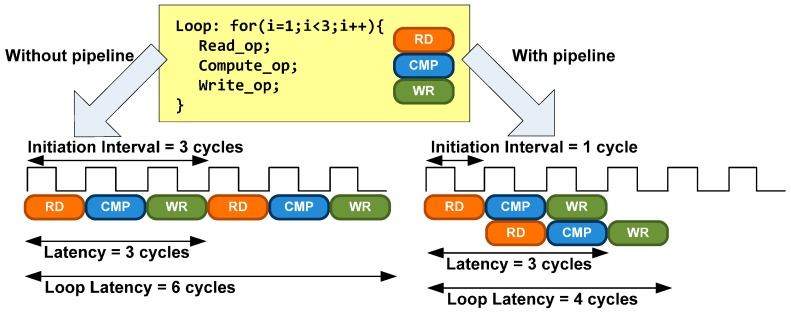
Loop pipelining scheme. Loop pragma reduce in two cycles the loop latency.

Each function has been analyzed in order to implement it in hardware according to the design methodology explained in the previous section (see Figure 7 and Figure 8). 

Pragmas or directives on the C code were used in SDSoC to achieve hardware performance optimization. They implemented architecture using pipelining, dataflow, unrolling and array partitioning. Pragmas are used to explore micro-architectures that satisfy the desired performance and chip area goals. Three pragmas have been used in the NFFT functions.

Loop unrolling pragma has been used in the for-loops for the three functions to unroll and create multiple independent operations rather than a single collection of operators. It creates multiple copies of the loop body and adjusts the loop iteration counter accordingly, so that it can exploit more parallelism among these operations. More parallelism means more throughput and higher system performance. 

Loop pipelining pragma is another technique to exploit parallelism between loop iterations. It allows the operations in a loop to be implemented in a concurrent manner as shown in Figure 8. An Initiation Interval (II) must be specified. It is the number of clock cycles between the start times of consecutive loop iterations.

Sequential data access pragma has been used at the beginning of *deconv*, *fft* and *conv* functions. This directive allows that a hardware function admits streaming access for an array data transfer (that is, each element is accessed precisely once in the index order). It avoids the implementation of a shared memory model for hardware function arguments (normally, hardware function calls involve copy-in, copy-out semantics for function arguments). This pragma is necessary because the latency to external Double Data Rate (DDR) memory from the programmable logic is significantly high and undesirable. 

## 5. Analysis, Results and Comparative Study

The originality of the implemented solution consists in the use and selection of an adequate set of pragmas. The use of pragmas permits the SDSoC tool to achieve an automatic optimization of the resulting hardware in FPGA. We have used profiling tools to detect the algorithm bottlenecks and to identify what parts of the code need to be improved. After the analysis of the profiling output, the pragmas were placed manually on the source code. The profiling tool that we have used is the SDSoC profiling library, *sds* *lib*, which allows us to identify the most CPU-intensive portions of our program.

In order to evaluate the improvement of the NFFT algorithm, we have implemented, independently for each function, a software solution and hardware solution. Once these solutions were analyzed, we evaluated the whole NFFT algorithm in the same way. The NFFT window function φ has been the Gaussian function. The Oversampling factor σ was set to 2 and the Cut-off parameter m was set to 6.

Table 1 shows the computational results of the SW and HW solutions for the deconvolution module. Results are provided for CPU cycles with different data sizes. The table shows that the hardware solution improves the software solution leading to a speed-up of 37 for a data length of 1024.

We repeated this process for the FFT module (Table 2), obtaining a significant improvement between the HW and SW solution. In this case, we obtained a speed-up of 380 for a data length of 1024. However, the results are different for the convolution module; the HW solution (Table 3) does not improve the SW solution considerably (speedup of 1.2). As we can see, a constant value of this magnitude is obtained independently from the number of samples. There is no simple way to improve these results since the subdivision of this module in minor parts would produce the same speed-up. To obtain better results, it is necessary to use a low level encoding of the module. This aspect is not considered in this paper since our goal was to explore the use of high level tools to obtain a fast and simple development of the desired hardware solution. 

This can be explained considering the pattern of memory access. The SDSoC provides a mechanism to allocate contiguous memory in physical address space using *sds alloc* and *sds free*. However, the convolution in Equation (13) is highly irregular. This causes multiple non-contiguous memory access and, consequently, the speed of the hardware coprocessor is significantly reduced.

Table 4 depicts the improvement when the three modules were selected to be accelerated in hardware. For each input data length, the hardware solution improves the software solution, obtaining speed-ups in the range [1.8, 4.07]. In this case, the speed-up from the worst module (convolution module) compensates the gain of the other two modules.

## 6. Conclusions

In this paper, we have presented an efficient NFFT implementation using hardware coprocessors. It uses an All-Programmable System-on-Chip (APSoC), a hybrid device with an ARM Processing System with Programmable Logic for high-performance digital signal processing through parallelism and pipelining techniques. The hardware accelerated NFFT algorithm into the PL, communicates with the CPU and external memory through an automatically-generated, application-specific data network comprised of Direct Memory Access (DMAs) and AXI interconnections.

The use of the SDSoC profiling tool and pragmas allowed us to detect the algorithm bottlenecks and to improve the speed-up of the algorithm. The use of the SDSoC environment provided us an embedded C/C++ application programming capability including an easy-to-use Eclipse-IDE and a comprehensive development platform. SDSoC included a full-system optimizing C/C++ compiler, system-level profiling and hardware/software event tracing, automated software acceleration in programming logic, automated generation of SW-HW connectivity, and integration with libraries to ease programming. The SDSoC compiler transformed function code into a complete hardware/software solution based on a user-specified target platform. However, hardware design knowledge was still necessary in order to interpret the results effectively.

The computational results with this technology show a considerable improvement when it is possible to use data streaming interface. This is the case of the deconvolution and FFT modules. Using streaming, it is possible to obtain speed-ups that reach 380 in the FFT module. However, when memory access is irregular as in the convolution module, the improvement is less pronounced. In the NFFT, the loops in the convolution module have variable limits and non-contiguous RAM access patterns. For this reason, the data motion network circuit between the PS and the PL accelerator that the SDSoC tool implements automatically does not choose the most optimal interface.

Our main goal in this work was to explore and test the capabilities of SDSoC without the need to perform a low level encoding. Results show that the obtained HW and SW solution always improves the SW solution. However, in some cases, it is necessary to use low level encoding to obtain better results. It would be interesting, as a possible future development, to go deeper into the convolution module, either by applying new techniques of parallelism and other algorithms based on different interpolation kernels. In addition, we are planning to use the obtained solution in several computational photography applications including the processing of lightfield images. 

## Figures and Tables

**Table 1:** Acceleration of the deconvolution function.

Size of Samples	Software Solution			Hardware Solution			Speed-Up Improvement
	CPU Cycles	% PS ^1^	% PL ^2^	CPU Cycles	% PS	% PL	
32 (2^5^)	265,328	24.6%	0.0%	14,652	4.1%	19.7%	18.11
64 (2^6^)	278,355	24.9%	0.0%	14,763	4.3%	20.0%	18.85
128 (2^7^)	304,407	25.3%	0.0%	14,983	4.5%	20.6%	20.32
256 (2^8^)	356,512	26.2%	0.0%	15,423	4.9%	21.7%	23.12
512 (2^9^)	460,722	27.8%	0.0%	16,303	5.7%	23.9%	28.26
1024 (2^10^)	669,142	31.1%	0.0%	18,064	7.4%	28.3%	37.04

^1^ Percentage of the Processing System (ARM) used. ^2^ Percentage of the Programmable Logic (FPGA) used.

**Table 2:** Acceleration of the FFT function.

Size of Samples	Software Solution			Hardware Solution			Speed-Up Improvement
	CPU Cycles	% ARM (PS)	% PL	CPU Cycles	% ARM (PS)	% PL	
32 (2^5^)	252,298	25.2%	0.0%	7844	4.1%	23.4%	32.16
64 (2^6^)	342,447	25.5%	0.0%	7849	4.3%	23.8%	43.63
128 (2^7^)	522,744	25.9%	0.0%	7859	4.5%	24.6%	66.52
256 (2^8^)	883,339	26.7%	0.0%	7880	4.9%	26.2%	112.10
512 (2^9^)	1,604,528	28.4%	0.0%	7919	5.7%	29.4%	202.62
1024 (2^10^)	3,046,906	31.9%	0.0%	7998	7.4%	35.7%	380.96

**Table 3:** Acceleration of the convolution function.

Size of Samples	Software Solution			Hardware Solution			Speed-Up Improvement
	CPU Cycles	% ARM (PS)	% PL	CPU Cycles	% ARM (PS)	% PL	
32 (2^5^)	159,932	61.3%	0.0%	143,336	4.1%	54.5%	1.12
64 (2^6^)	348,085	62.3%	0.0%	311,964	4.3%	55.1%	1.12
128 (2^7^)	724,390	64.1%	0.0%	649,220	4.5%	55.9%	1.12
256 (2^8^)	1,476,999	67.8%	0.0%	1,323,732	4.9%	57.7%	1.12
512 (2^9^)	2,982,218	75.2%	0.0%	2,682,756	5.7%	61.2%	1.11
1024 (2^10^)	5,992,656	89.9%	0.0%	5,370,804	7.4%	68.3%	1.12

**Table 4:** Acceleration of the whole system.

Size of Samples	Software Solution			Hardware Solution			Speed-Up Improvement
	CPU Cycles	% ARM (PS)	% PL	CPU Cycles	% ARM (PS)	% PL	
32 (2^5^)	678,094	69.9%	0.0%	166,582	4.1%	61.5%	4.07
64 (2^6^)	969,428	70.7%	0.0%	335,397	4.3%	62.5%	2.90
128 (2^7^)	1,552,094	72.2%	0.0%	673,027	4.5%	64.4%	2.31
256 (2^8^)	2,717,426	75.3%	0.0%	1,348,287	4.9%	68.6%	2.02
512 (2^9^)	5,048,090	81.4%	0.0%	2,698,807	5.7%	76.9%	1.87
1024 (2^10^)	9,709,418	93.6%	0.0%	5,399,846	7.4%	91.3%	1.80

## References

[1] Cooley J.W., Tukey J.W. (1965). An algorithm for machine calculation of complex Fourier series. Math. Comput..

[2] Renganarayana L., Rajopadhye S. An approach to SAR imaging by means of non-uniform FFTs. Proceedings of the IEEE International Geoscience and Remote Sensing Symposium.

[3] Pérez F., Pérez A., Rodríguez M., Magdaleno E. (2015). A fast and memory-efficient Discrete Focal Stack Transform for plenoptic sensors. Digit. Signal Process..

[4] Knopp T., Kunis S., Potts D. (2007). A note on the iterative MRI reconstruction from nonuniform k-space data. Int. J. Biomed. Imaging.

[5] Ying S., Kuo J. (2005). Application of two-dimensional nonuniform fast Fourier transform (2-d NuFFT) technique to analysis of shielded microstrip circuits. IEEE Trans. Microw. Theory Tech..

[6] Duijndam A.J.W., Schonewille M.A. (1999). Nonuniform fast Fourier transform. Geophysics.

[7] Pérez J., Magdaleno E., Pérez F., Rodríguez M., Hernández D., Corrales J. (2014). Super-Resolution in plenoptic cameras using FPGAs. Sensors.

[8] Pérez F., Pérez A., Rodríguez M., Magdaleno E. (2015). Super-resolved Fourier-slice refocusing in plenoptic cameras. J. Math. Imaging Vis..

[9] O’Sullivan J. (1985). A fast sinc function gridding algorithm for Fourier inversion in computer tomography. IEEE Trans. Med. Imaging.

[10] Scramek R., Schwab F., Perley R.A., Schwab F.R., Bridle A.H. (1988). Imaging. Synthesis Imaging in Radio Astronomy: A Collection of Lectures from the Third NRAO Synthesis Imaging Summer School.

[11] Schomberg H., Timmer J. (1995). The Gridding method for image reconstruction by Fourier transformation. IEEE Trans. Med. Imaging.

[12] Potts D., Steidl G., Tasche M., Benedetto J., Ferreira P. (2001). Fast Fourier transforms for nonequispaced data: A tutorial. Modern Sampling Theory: Mathematics and Applications.

[13] Dutt A., Rokhlin V. (1993). Fast Fourier transforms for nonequispaced data. SIAM J. Sci. Comput..

[14] Beylkin G. (1995). On the fast Fourier transform of functions with singularities. Appl. Comput. Harmon. Anal..

[15] Steidl G. (1998). A note on fast Fourier transforms for nonequispaced grids. Adv. Comput. Math..

[16] Fessler J.A., Sutton B.P. (2003). Nonuniform fast Fourier transforms using min-max interpolation. IEEE Trans. Signal Process..

[17] Keiner J., Kunis S., Potts D. (2009). Using NFFT 3—A software library for various nonequispaced fast Fourier transforms. ACM Trans. Math. Softw..

[18] Kunis S., Potts D. (2002). NFFT, Software Package, C Subroutine Library.

[19] Fessler J.A., Sutton B.P. (2002). NUFFT—Nonuniform FFT Toolbox for Matlab. http://web.eecs.umich.edu/~fessler/code/index.html.

[20] Underwood K., Hemmert K. Closing the gap: CPU and FPGA trends in sustainable floating—Point BLAS performance. Proceedings of the 12th Annual IEEE Symposium on Field-Programmable Custom Computing Machines.

[21] Altera Corporation (2009). FPGA Coprocessing Evolution: Sustained Performance Approaches Peak Performance.

[22] Kestur S., Park S., Irick K.M., Narayanan V. Accelerating the nonuniform fast fourier transform using FPGAs. Proceedings of the 2010 18th IEEE Annual International Symposium on Field-Programmable Custom Computing Machines (FCCM).

[23] Jackson J.I. (1991). Selection of a convolution function for Fourier inversion using gridding. IEEE Trans. Med. Imaging.

[24] Avnet (2014). Zedboard (Zynq Evaluation and Development) Hardware User’s Guide. Version 2.2. http://zedboard.org/sites/default/files/documentations/ZedBoard_HW_UG_v2_2.pdf.

[25] Xilinx (2016). Zynq-7000 All Programmable SoC. Technical Reference Manual. UG585 (v1.11). https://www.xilinx.com/support/documentation/user_guides/ug585-Zynq-7000-TRM.pdf.

[26] ARM (2012). Cortex-A9 MPCore Technical Reference Manual. Revision r4p1. https://static.docs.arm.com/ddi0407/i/DDI0407.pdf.

[27] Xilinx (2016). Zynq-7000 All Programmable SoC Overview. DS190 (v1.10). https://www.xilinx.com/support/documentation/data_sheets/ds190-Zynq-7000-Overview.pdf.

[28] Chapman K. (2014). PicoBlaze for Spartan-6, Virtex-6, 7-Series, Zynq and UltraScale Devices (KCPSM6). https://www.xilinx.com/ipcenter/processor_central/picoblaze/member/.

[29] Xilinx (2012). LogiCORE IP MicroBlaze Micro Controller System (v1.1). DS865. https://www.xilinx.com/support/documentation/sw_manuals/xilinx14_1/ds865_microblaze_mcs.pdf.

[30] Crockett L.H., Elliot R.A., Enderwitz M.A., Stewart R.W. (2014). Designing with Zynq. The Zynq Book: Embedded Processing with the ARM Cortex-A9 on the Xilinx Zynq-7000 All Programmable SoC.

[31] Xilinx (2016). Vivado Design Suite User Guide. Using the Vivado IDE. UG893 (v2016.3). https://www.xilinx.com/support/documentation/sw_manuals/xilinx2016_3/ug893-vivado-ide.pdf.

[32] Xilinx (2016). Xilinx Software Development Kit (SDK) User Guide. UG782 (V2016.2). https://www.xilinx.com/html_docs/xilinx2016_2/SDK_Doc/index.html.

[33] Xilinx (2016). SDSoC Environment User Guide. UG1027 (v2016.2). https://www.xilinx.com/support/documentation/sw_manuals/xilinx2016_2/ug1027-sdsoc-user-guide.pdf.

